# IFN-I Mediates Dysfunction of Endothelial Progenitor Cells in Atherosclerosis of Systemic Lupus Erythematosus

**DOI:** 10.3389/fimmu.2020.581385

**Published:** 2020-11-11

**Authors:** Xuewei Ding, Wei Xiang, Xiaojie He

**Affiliations:** ^1^ Institute of Pediatrics, The Second Xiangya Hospital, Central South University, Changsha, China; ^2^ Laboratory of Pediatric Nephrology, Institute of Pediatrics, The Second Xiangya Hospital, Central South University, Changsha, China; ^3^ Key Laboratory of Tropical Translational Medicine of Ministry of Education, Hainan Medical University, NHC Key Laboratory of Control of Tropical diseases (Hainan Medical University), Haikou, China

**Keywords:** atherosclerosis, endothelial cell, endothelial progenitor cell, pathogenesis, systemic lupus erythematosus, IFN-I

## Abstract

Systemic lupus erythematosus (SLE) is a multi-system autoimmune disease including the cardiovascular system. Atherosclerosis is the most common cardiovascular complication of SLE and a significant risk factor for morbidity and mortality. Vascular damage/protection mechanism in SLE patients is out of balance, caused by the cascade reaction among oxidative stress, proinflammatory cytokines, Neutrophil Extracellular Traps, activation of B cells and autoantibodies and abnormal T cells. As a precursor cell repairing vascular endothelium, endothelial progenitor cells (EPCs) belong to the protective mechanism and show the reduced number and impaired function in SLE. However, the pathological mechanism of EPCs dysfunction in SLE remains ill-defined. This paper reviews the latest SLE epidemiology and pathogenesis, discusses the changes in the number and function of EPCs in SLE, expounds the role of EPCs in SLE atherosclerosis, and provides new guidance and theoretical basis for exploring novel targets for SLE treatment.

## Introduction

SLE is an immune complex-mediated autoimmune disease involving multiple systems. Its prevalence and incidence rate can be as high as 241/100,000 per year and 23.2/100,000 per year, and the rate of premature death is 2–3 times that of healthy people (1). Since 2000, the prevalence rate of adult SLE in women has been 30–150/100,000, and the incidence rate is 2.2–23.1/100,000 per year (2). SLE is also an autoimmune disease characterized by cardiovascular disease (CVD). A multicenter study found that a quarter of the nearly 10,000 deaths from SLE were caused by CVD (3). Current studies have demonstrated that the inherent factors of SLE are independent risk factors for the premature occurrence of atherosclerosis in SLE patients (4). With the improvement of the diagnosis and treatment, the early mortality of SLE patients has been dramatically reduced. However, atherosclerosis is still one of the leading causes of death of late SLE patients. It is of considerable significance to explore the natural course and mechanism of SLE combined with atherosclerosis, find useful therapeutic targets, provide evidence for clinical intervention, and delay the death of SLE.

Vascular endothelial dysfunction is the starting point in SLE atherosclerosis. Endothelial progenitor cells (EPCs) are closely related to vascular endothelial function. Therefore, the relationship between atherosclerosis and EPCs in SLE is a research direction worth exploring. However, in recent decades, there are few studies on the relationship between atherosclerosis and EPCs in SLE, and the results are controversial. This paper analyzes the changes in the number and function of EPCs in SLE and reviews the potential role of EPCs in SLE atherosclerosis.

## Mechanism of Atherosclerosis in SLE

**Figure 1:**
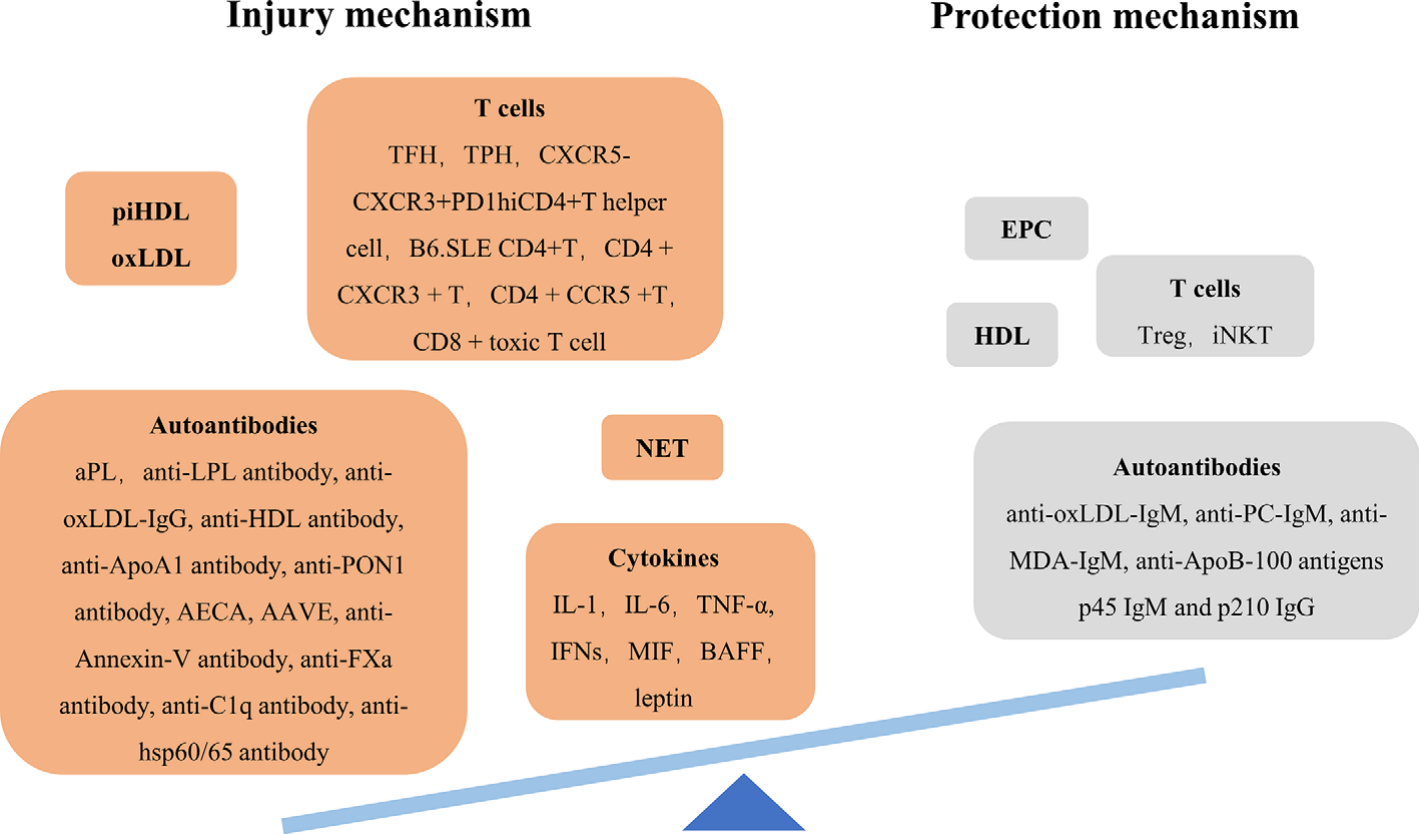
Imbalance of injury/protection mechanism of SLE arteriosclerosis. AAVE anti-vascular endothelial-cadherin antibody AECA anti-endothelial cell antibody aPL antiphospholipid antibody BAFF B cell-activating factor EPC endothelial progenitor cell iNKT invariant natural killer T cell LPL lipoprotein lipase MDA malondialdehyde; MIF, macrophage migration inhibitory factor; NET, neutrophil extracellular trap; PC, choline phosphate; TFH, follicular helper T cell; TPF, peripheral helper T cell.

Arteriosclerosis is a series of aggregation events of leukocytes and vascular smooth muscle cells (VSMCs) in intima triggered by endothelial dysfunction and lipoprotein retention, resulting in fibrous plaques. Then fibrous plaques rupture, followed by thrombosis. This process requires the immune response’s help (5, 6). The abnormal immune response driven by SLE enhances vascular injury mechanism and weaken repair mechanism, breaking vascular dynamic balance which determines the occurrence of CVD (**Figure 1**).

### Oxidative Stress

Mitochondrial dysfunctions, abnormal bioenergetics/immunometabolism and telomere/telomerase disequilibrium endowed SLE patients with intense oxidative stress (7). Among the three main targets of oxidative stress, oxidized lipids—oxLDL and proinflammatory HDL (piHDL)—play a prominent role in accelerating SLE atherosclerosis (8). OxLDL participates in many stages of atherosclerosis, from endothelial dysfunction to plaque rupture (6, 9). Normal HDL plays a role in protecting atherosclerosis by promoting cholesterol outflow, inhibiting vascular inflammation and scavenging oxidizing substances. However, lupus-altered HDL shifts from a normal anti-inflammatory state to a proinflammatory state, causing atherosclerosis (10). Increased piHDL weakens the ability to prevent LDL oxidation (8).

### Cytokines

**Figure 2:**
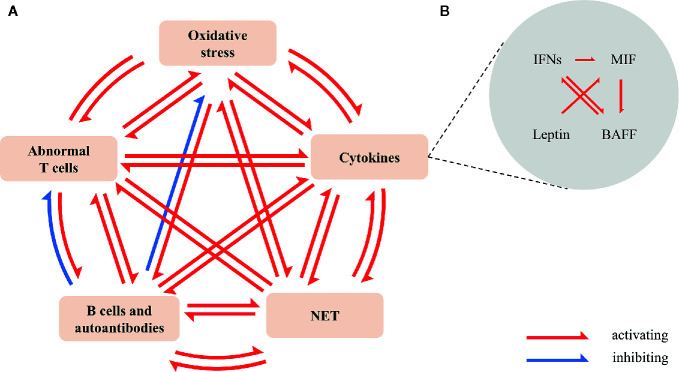
Cross-talk between oxidative stress, cytokines, NETs, activation of B cells and autoantibodies, and abnormal T cells in SLE. **(A)** Oxidative stress promotes the production of IFN-I (11), NETs (12), autoantibodies (13), and the imbalance of Th17/Treg (14). IFN-α and IFN-γ promote lipids oxidative modification (15, 16); BAFF promotes the production of autoantibodies (17), the release of NETs (18) and the activation of T cells (19); leptin promotes the production of autoantibodies, the release of NET and the imbalance of Th17/Treg (20, 21). NET encourages oxidation HDL (22), the expression of IFN-α (23) and IL-1β (24), and activates NET-specific memory B cells to proliferate and secrete polyclonal IgG (25). Overactive T cells increase ROS (26) and cytokines, especially IFN-γ; TFH (27, 28), CXCR5-CXCR3^+^PD1hiCD4^+^T helper cell (29), and peripheral helper T cell (TPH) (30) promote the differentiation of B cells and the production of antibodies. SLE-related autoantibodies and immune complexes induce the release of NET (31); anti-ApoA1-IgG guides the expression of cytokines (32). Anti-PC-IgM increases Tregs (33); anti-PC-IgM and anti-MDA-IgM reduce oxidative stress (34). **(B)** IFN-I (35, 36) and IFN-II (37) induce the expression and mobilization of BAFF. BAFF promotes the activation of B cells by IFN (38). Moreover, IFN-I encourages the production of MIF (39). MIF/CD74 signal regulates BAFF (40, 41). Leptin enhances MIF-induced inflammation (42). Besides, IFNs, MIF and leptin strengthen the expression of chemokine, adhesion molecule, TNF- α and ILs. BAFF, B cell-activating factor; MDA, malondialdehyde; MIF, macrophage migration inhibitory factor; NET, neutrophil extracellular trap; PC, choline phosphate; ROS, reactive oxygen species; TFH, follicular helper T cell; TPF, peripheral helper T cell.

Cytokines, the primary regulators of immune responses, regulate and coordinate multiple stages of atherosclerosis. There is a cascade reaction between these proinflammatory cytokines in accelerating SLE atherosclerosis (**Figure 2**).

IFNs are divided into three classes: IFN-I (IFN-α, IFN-β, IFN-δ, IFN-ϵ, IFN-κ, IFN-τ, IFN-ω), IFN-II (IFN-γ), IFN-III (IFN-λ1, IFN-λ2, IFN-λ3). IFNs participated in the whole process of atherosclerosis, especially IFN-I (15, 43–45). For example, IFN-α and IFN-γ promote lipoproteins’ oxidation (15, 16). IFN-α promotes endothelial dysfunction by accelerating endothelial cells (ECs) apoptosis and damaging EPCs, one of the vascular repair mechanisms (15, 46–53). IFN-α enhances the expression of chemokine and adhesion molecules without leukocytes adhesion (53); while IFN-γ can regulate the attraction and adhesion of leukocytes (54). IFN-α induces the up-regulation of SR-A expression in monocytes/macrophages, then promoting the lipid uptake and the formation of macrophage-derived foam cells (55); IFN-γ not only up-regulates SR-A, but also up-regulate ACAT1 (56) and inhibit specific anti-atherosclerotic MSRN proteins (APOE and C3) in macrophages (57) to reduce cholesterol efflux. IFN-α prevents smooth muscle progenitor cell (SMPC) from maturation which could give rise to macrophages and eventually foam cells (58); IFN-γ enhances VSMCs’ proliferation and migration (56). IFN-α and IFN-γ induce VSMC and macrophages apoptosis in atherosclerotic plaques, contributing to plaque instability (59–61). Moreover, IFN-α inhibits the expression of type I collagen gene COL1A1 in VSMCs (62) and induces the synthesis of TNF-α, IL-12 and MMP-9 (63); while IFN-γ inhibits the expression of type I collagen gene COL1A2 in VSMCs (64) and induces the synthesis of MMP-1, MMP-2 and MMP-9 (56). Besides, IFN-α forms an IFN-α-platelet-CD154-CD40 forward feedback loop to promote thrombosis (65, 66).

Macrophage migration inhibitory factor (MIF) is an inflammatory and chemokine-like cytokine and an upstream regulator of innate immunity. MIF enhances LDL uptake (67), recruits monocytes and T cells (68–70), migrates VSMCs (71), resulting in plaques. MIF also increases the expression of MMP-1 and MMP-9, inducing plaques rupture (72, 73).

B-Cell Activating Factor (BAFF) is a critical factor in B cell maturation, survival and function, and an independent factor in accelerating SLE atherosclerosis (17). BAFF/BAFF-R axis supports pathogenic B cells producing pathogenic anti-IgG-oxLDL antibodies (74, 75), which is over-activated in SLE (76). The co-expression of BAFF/TNFSF13B and APRIL/TNFSF13 in the plaque lymphocytes and macrophages up-regulate FURIN, the primary Proprotein convertase subtilisin/Kexin (PCSK), which inactivates lipases and regulates inflammation in atherosclerosis (19). And BAFF weakens EPCs’ function and promotes EPCs’ apoptosis (77).

As an immunopotentiator (78), leptin significantly increases the risk of atherosclerosis in SLE patients (79). And the serum leptin level ≥ 34ng/dL was significantly correlated with carotid plaque (79). Leptin induces oxidative stress, increases MCP-1, TNF-α, IL-6 and endothelin-1, accompanied by the expression of other EC adhesion molecules, MMPs and VEGF, which damages VSMCs and ECs (80). And leptin promotes the secretion of atherosclerotic factor (42, 81). Besides, leptin promotes the production of autoantibodies, increases the release of NET and imbalance of Th17/Treg in SLE (20).

### Neutrophil Extracellular Traps (NETs)

NET is a unique form of neutrophils death, characterized by the extrusion of chromatin and a driver of SLE atherosclerosis (82–87). NETs damages ECs. They promote vascular leakage and endothelial-to-mesenchymal transition through the degradation of VE-cadherin and the activation of β-catenin signaling (87); they induce EC death through the activation of endothelial MMP-2 (88). NETs also kill VSMCs (89). Moreover, NETs mediate HDL’s oxidation, interfering with cholesterol outflow (22). NETs induce the secretion of IFN-α (23) and IL-1β (24). Serine proteases from NETs degrade tissue factor pathway inhibitor (TFPI) (90) and promote FXII (91) that activate coagulation cascade and thrombosis.

### The Activation of B Cells and Autoantibodies

B cells mainly affect atherosclerosis by producing autoantibodies: B1 cells secrete protective natural IgM and IgA antibodies, whereas B2 cells produce pathogenic IgG antibodies. And the tendency of overactive B cells to produce pathogenic IgG antibodies is a potential risk factor for lupus-associated atherosclerosis (17). In particular, antiphospholipid antibodies (aPL) have been identified as independent predictors of atherosclerotic plaque progression in SLE (92, 93).

Anti-HDL-IgG induces LDL to enter the ECs, which is a major contributor to atherosclerosis in SLE. Recently, Kurien BT et al. found that SLE RNP and anti-Ro/LaRNP antibodies probably increase the level of anti-oxLDL antibodies (94). Anti-HDL antibody, anti-ApoA1 antibody and anti-PON1 antibody probably have a common atherogenic pathway—they unbalance PON-1/MPO, which enhances lipids oxidative modification and interferes with HDL’s anti-inflammation (95–97). Besides, anti-ApoA1-IgG has two pathways that induce atherosclerosis in a TLR2/TLR4/CD14-dependent manner: it activates transcriptional nuclear factor NF-kB to guide the expression of inflammatory factors; it provides an alternative (or a concomitant) signal to PI3K in an Src-dependent pathway, activates L-type Ca2^+^ channels and potassium/calcium exchangers, resulting in the depolarization of myocardial plasma membrane (32). Anti-FXa-IgG unbalances hemostasis and thrombosis by inhibiting the FXa enzyme (98) and promotes endothelial dysfunction by enhancing FXa-PAR-mediated Ca2^+^ signal transduction (99). Recent studies have found that IgA-AECA is involved in SLE endothelial damage by recognizing the membrane proteins of ECs (100). Anti-C1q antibody plays a role in atherosclerosis by reducing C1q’s level and protective effects (101, 102), which polarizes macrophages towards an M2-like anti-inflammatory phenotype (103) and improves macrophages’ survival and excretion (104).

There are potential protective autoantibodies in SLE patients, such as anti-oxLDL-IgM, anti-ApoB100 antibodies, anti-choline phosphate (PC) antibodies and anti-malondialdehyde (MDA) antibodies. The first three have a synergistic effect: they reduce the level of oxLDL, the uptake of oxLDL, and the formation of foam cells (105–107). And Anti-PC-IgM increases Tregs in SLE and atherosclerosis, reduces IL-17 and TNF-α, and makes dendritic cells (DCs) immature (33). The combined application of anti-PC-IgM and anti-MDA-IgM has a doubly preventive impact on atherosclerosis (34). However, SLE patients showed a low level of protective autoantibodies (34, 107). Some dietary and metabolic factors may be responsible for the low levels of anti-PC-IgM and anti-MDA-IgM (108).

SLE increases the risk of CVD by promoting pathogenic autoantibodies and inhibiting potential protective autoantibodies.

### The Abnormal T Cells

Abnormal T cell subsets are considered to be an essential factor leading to endothelial dysfunction and CVD in SLE patients. Tregs are protective T cells in atherogenesis, inhibiting atherogenic T cell subsets and inflammation. And Treg/Th17 imbalance is common in SLE, becoming a risk factor for atherosclerosis (109). In human circulation, atherosclerosis’s severity is not directly related to the number of Tregs (110) but is closely related to the dysfunction of Tregs (111). During atherosclerosis, most Treg lost Foxp3 expression and its immunosuppressive function, then transform into follicular helper T cell (TFH) (112), which is used to stimulate the formation of germinal center (GC) and the selection of high-affinity B cells in GC (27). TFH has also been shown to accelerate atherosclerosis, although not necessarily by inducing the production of pathogenic IgG (112, 113). Besides, CD4^+^T cells in peripheral blood of SLE patients highly express CCR5 and CXCR3 promoting the migration of inflammatory T cells to the arterial wall in a chemokine-dependent way (114, 115). In particular, CCR5 is the critical factor for CD4+T cells homing to atherosclerotic plaques (116).

A recent study has shown that Invariant natural killer T (iNKT) in SLE patients has an anti-atherosclerotic phenotype which induces macrophages to polarize into anti-inflammatory and anti-atherosclerotic M2 phenotype (117). The protection is triggered in early atherosclerosis but is lost or submerged in the development of clinical atherosclerosis (117).

Oxidative stress, cytokines, NETs, activation of B cells and autoantibodies, and abnormal T cells in SLE interact with each other, amplifying their pro-atherogenic effects (**Figure 2**). As a result, the dynamic vascular homeostasis is broken in SLE patients, characterized by enhanced injury mechanism and weakened protection mechanism. Subclinical atherosclerosis in SLE accelerates, even in environments with low disease activity (92).

## The Role of EPCs in Arteriosclerosis

Atherosclerosis is a manifestation of the imbalance between vascular injury and protection mechanisms, especially in endothelial dysfunction. EPCs are the primary protection mechanism for endothelial dysfunction, which promote angiogenesis and maintains endothelial integrity with a series of reactions. But the situation of this protection mechanism in SLE is not optimistic.

### Classification, Immunophenotype, and Physiology of EPCs

Scientists have reached a consensus that EPCs isolated by cell culture are distinguished into two different groups: myeloid angiogenic cells (MACs), used to identify early EPCs (118); endothelial colony forming cells (ECFCs), used to identify late EPCs (119). They promote vascular repair through different mechanisms (120). ECFCs, considered to be real EPCs, can differentiate into ECs promoting vascular repair and neovascularization (121), with the immunophenotype positive for CD31, CD105, CD146, and negative for CD45, CD14 (120). MACs can’t become ECs but secretes angiogenic cytokines to promote angiogenesis through a paracrine mechanism (122), with the immunophenotype positive for CD45, CD14, CD31, and negative for CD146, CD133, and Tie2 (120) (**Table 1**).

**Table 1:** Classification, immunophenotype and physiology of EPCs.

Classification	Physiology	Immunophenotype
MACs	Secreting angiogenic cytokines	Positive : VEGFR2,CD133,CD45,CD115,CD14,CD31 Negative : CD146, CD34,Tie2
ECFCs	Differentiating into ECs	Positive : VEGFR2,CD34,C D31,CD105,CD146 Negative : CD133,CD45, CD115,CD14

### The Role of EPCs in Vascular Repair

After the injury, vascular repair occurs by accelerating the replacement of ECs. Re-endothelialization is a self-repairing process that maintains vascular endothelial protection after injury, including the proliferation and migration of adjacent intact ECs, resident EPCs and recruited EPCs. EPCs provide an endogenous repair mechanism to counteract persistent cell damage induced by risk factors. Scientists suggested EPCs are a useful tool for the treatment of endothelial injury in regenerative cardiovascular medicine (123–126). Thus, EPCs have been studied as biomarkers for the diagnosis and prognosis of CVD (127–129).

#### ECs

Healthy ECs protect atherosclerosis by promoting vasodilation, antioxidant and anti-inflammatory and inhibiting leukocyte adhesion and migration, and smooth muscle cell proliferation and migration. Remarkably, ECs can repair themselves. VEGF activates Cdc-42 and Rac1, mediates the formation of filamentous pseudopodia and plate pseudopodia, leading to EC migration (130). SDF-1 activates GPCR-dependent p110γPI3K, increases the expression of FoxM1 in ECs, participates in the transcriptional regulation of cell cycle progression genes, promoting the proliferation of ECs (131). Also, FoxM1 promotes re-adhesion between ECs through transcriptional control of β-catenin (132). When cells exfoliate after injury, surrounding ECs proliferate and migrate to coverage the basement membrane. However, mature ECs have limited ability to replace damaged ECs. Compared to ECs, EPCs show a higher proliferation potential, thus can serve as an additional source of ECs.

#### EPCs

**Figure 3:**
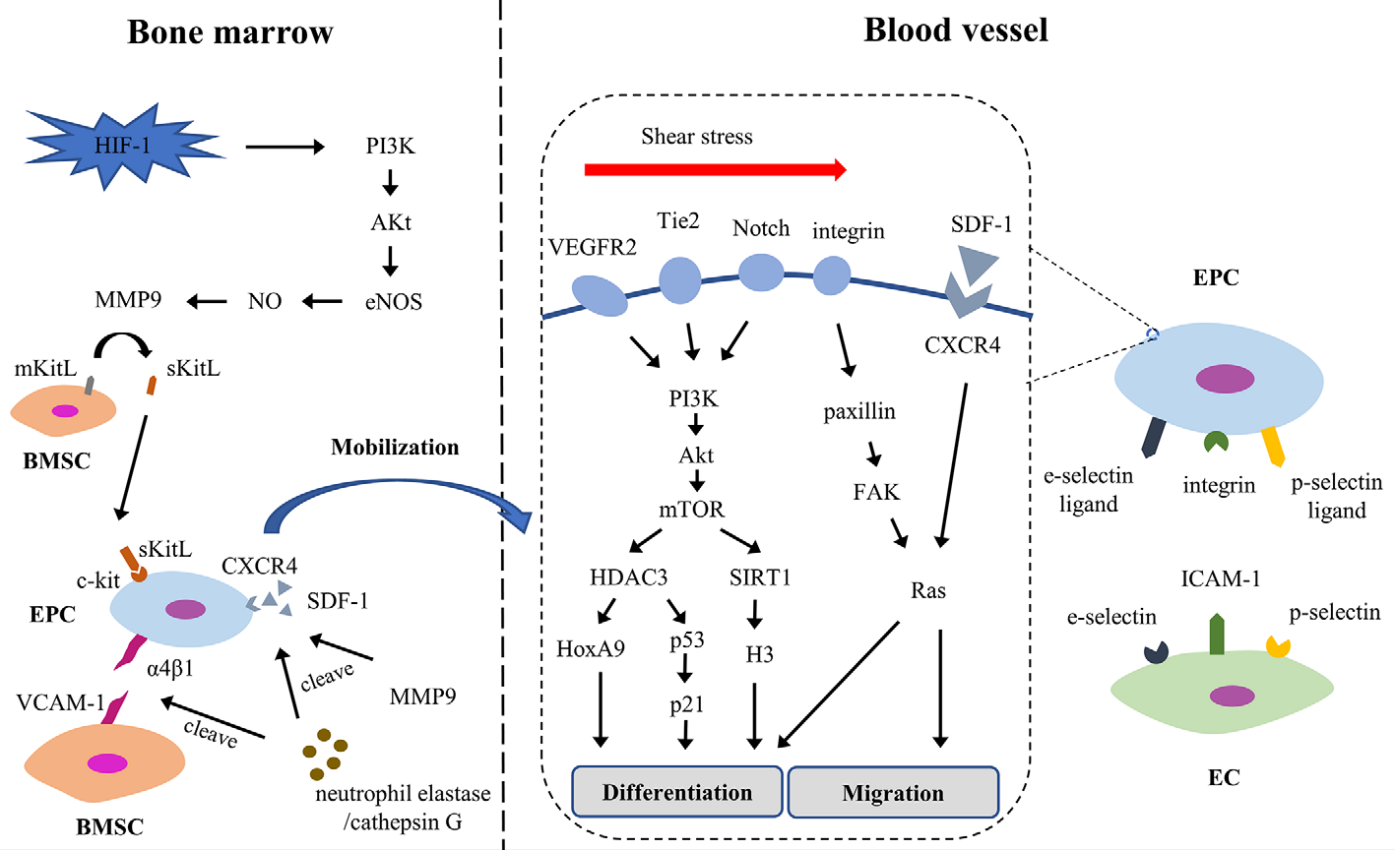
The process of EPC re-endothelialization. Akt, Protein kinase B; BMSC, bone marrow stromal cell; CXCR4, chemokine (C-X-C motif) receptor 4; EC, endothelial cell; EPC, endothelial progenitor cell; eNOS, endothelial nitric oxide synthase; FAK, focal adhesion kinase; HDAC3, histone deacetylase 3; HIF-1, hypoxia-inducible factor 1; HoxA9, Homeobox A9; ICAM-1, intercellular adhesion molecule-1; mKitL, membrane-bound form of Kit ligand; MMP9, matrix metalloproteinase-9; mTOR, mammalian target of rapamycin; PI3K, phosphatidylinositol-3-kinase; SDF-1, stromal cell-derived factor-1; sKitL, soluble Kit-ligand; VCAM-1, vascular cell adhesion molecule-1; VEGFR2, vascular endothelial growth factor receptor-2.

EPCs could differentiation into ECs. EPCs locate at the site of vascular injury, restore endothelial integrity and participate in neovascularization. The process of re-endothelialization includes mobilization, chemotaxis, homing, proliferation and differentiation (**Figure 3**). Early EPCs release growth factors, adhesion molecules and chemokines to promote the proliferation, survival and migration of late EPCs; late EPCs directly participate in the formation of endothelium (133). EPCs also release exocrine bodies to respond to injured ECs (134, 135).

##### Mobilization

The mobilization is the first step and is strictly regulated. EPCs are mainly seen in the bone marrow and in an inactive state which bind to bone marrow stromal cells (BMSCs) through the interaction of integrins (α4β1 and β3) and VCAM-1 (136). Under hypoxia, hypoxia-inducible factor 1 (HIF-1) rapidly increases, then weakens the interaction between EPCs and BMSC through NO and MMP-9 in PI3K/AKt/eNOS-dependent manner. Moreover, neutrophil elastase and cathepsin G prevent EPCs from combining with BMSCs by cutting integrin and VCAM-1; and they cooperated with MMP9 to degrade SDF-1 in peripheral blood matrix niches forming a high SDF-1 concentration gradient. Under the synergistic action of elastase, cathepsin G and MMPs, EPCs are driven into the peripheral circulation (137).

##### Homing

After entering the peripheral circulation from the bone marrow, EPCs are summoned and stay at the site of endothelial injury in the tissue. This process involves multi-step cascade adhesion and signaling events, including chemotaxis, involvement, adhesion and migration (138). The SDF-1/CXCR4 axis regulates the downstream signal Rac, changes the polarity and cytoskeleton of the cells, maintains the motor state of the transitional cells, and navigates the EPCs to the target organ (139). Meanwhile, integrin, p-selectin ligand and e-selectin ligand expressed on EPCs interact with p-selectin, e-selectin and ICAM-1 expressed on activated ECs, supporting EPCs adhesion and migration to ECs (140, 141). Some studies have shown that SDF-1 increases the expression of e-selectin in microvascular ECs and then increases the adhesion of EC-EPC (142).

##### Differentiation

On the way to the target organ, EPCs begin to differentiate into ECs. During differentiation, cytokines and shear stress trigger a series of events, which cause EPCs to acquire some phenotypic characteristics of ECs. Shear stress supports the differentiation and proliferation of EPCs *via* VEGFR2, Tie2, Notch, and β1/3 integrin signaling (143). It stabilizes and activates histone deacetylase 3 (HDAC3) through the VEGFR2/Tie2/Notch/PI3K/Akt/mTOR pathway, which in turn deacetylated p53, leading to increased cell cycle arrest protein p21 and endothelial markers (144). The homeobox transcription factor HoxA9 contributes to HDAC-mediated differentiation (145). Histone deacetylase SIRT1, another downstream factor of shear stress/PI3K/Akt pathway, is overexpressed in EPCs and decreases histone H3 acetylation, upregulating endothelial markers (146). Beside, integrins β1 and β3, also overexpressed, enhance the expression of endothelial markers *via* paxillin/FAK/RAS/ERK pathway (147–149).

Mobilized EPCs enter into the peripheral blood and build a cell pool, repairing the endothelium by forming a patch at the site of intimal injury. EPCs represent negative feedback in intravascular homeostasis. The number and function of EPCs are regulated by the same molecular pathway, so the decrease of EPCs number is related to weakened function, and the increase of EPCs number is related to enhanced function.

### Changes in the Number and Function of EPCs in SLE

There are 15 research articles about the number and function of SLE EPCs by searching “(Endothelial Progenitor Cells) AND (Lupus Erythematosus, Systemic)” in PubMed, which have shown inconsistent results (**Table 2**). Most of the results on the quantitative studies of SLE EPCs have shown a low level. Four studies have shown different results. The difference in the detection, quantification and identification of EPCs and the active phase of SLE might explain the quantitative differences. Studies on the qualitative of SLE EPCs also showed different results. Ablin JN et al. shown enhanced adhesion of SLE EPCs (156), while the others shown weakened proliferation/migration/adhesion/differentiation (46–49, 77, 150, 153, 154, 157–159). The different adhesion test and quantification seems to be the reason.

**Table 2:** Quantitative analysis of circulating EPCs between SLE and healthy control.

Results	Research objects	Surface labelings for the determination of EPCs	Detection methods	Quantization methods	References
Low level of EPCs in the SLE group	18 patients with SLE	CD34^+^ VEGFR2^+^	Flow cytometry Cell colony	Relative to the number of lymphocytes	(77)
	132 children with SLE	CD34^+^ CD133 ^+^	Flow cytometry	Absolute count per unit of blood	(150)
	90 patients with SLE	CD34^+^ VEGFR2^+^	Flow cytometry	Absolute count per unit of blood	(151)
	17 patients with SLE	CD34^+^ CD133^+^ VEGFR2^+^ /CD34^+^ VEGFR2^+^/CD133^+^ VEGFR2^+^	Flow cytometry	Absolute count per unit of blood	(152)
	70 patients with SLE	CD34^+^ VEGFR2^+^	Flow cytometry Cell colony	Absolute count per unit of blood	(47)
	135 patients with SLE	CD34^+^CD133^+^	Flow cytometry Cell colony	Absolute count per unit of blood	(48)
	44 patients with SLE	CD34^+^CD133^+^	Flow cytometry	Absolute count per unit of blood	(153)
	15 patients with SLE	CD34^+^VEGFR2^+^	Flow cytometry	Absolute count per unit of blood	(154)
	gld.apoE-/- mice	Sca-1^+^ VEGFR2^+^	Flow cytometry	Relative to the number of lymphocytes	(155)
	gld.apoE-/- mice	Sca-1^+^ VEGFR2^+^	Flow cytometry	Relative to the number of lymphocytes	(46)
	NZB/W mice	CD34^+^ VEGFR2^+^	Flow cytometry	Relative to the number of lymphocytes	(49)
No significant difference	31 patients with SLE	Tie-1^+^ VEGFR2^+^ CD31^+^	Cell colony	The number of colony	(156)
	35 patients with SLE	CD34^+^ VEGFR2^+^	Flow cytometry Cell colony	Relative to the number of lymphocytes	(157)
	31 patients with SLE	CD34^+^ VEGFR2^+^ CD 133^+^	Flow cytometry Cell colony	Relative to the number of lymphocytes	(158)
Low level of CD34+VEGFR2+ cells and high level of CD133+VEGFR2+ cells in the SLE group	19 patients with SLE	CD133^+^VEGFR2^+^ cells represent early EPCs, and CD34^+^VEGFR2^+^ cells represent late EPCs	Flow cytometry	Absolute count per unit of blood	(159)

### Causes of Reduced Number and Impaired Function of EPCs in SLE

Although the results are controversial, we believe that SLE EPCs show a trend of reduced number and impaired function. The risk factors (IFN-I, BAFF, OPG, IL-10, IL-18) and protective factors (Tang) both exist in SLE. The reduced number and impaired function of SLE EPCs seem to be the result of the game between the two sides.

There is no doubt that IFN-I accelerates SLE atherosclerosis, whether in the initiation or development of the disease (15, 52). The adult and mouse models’ researches conclude that IFN-I accelerating SLE atherosclerosis by interfering with EPCs (15, 46–49, 51, 52, 160). Like adult-onset SLE, childhood-onset SLE also shown reduced number and impaired function of EPCs (150). But there was no significant correlation between IFN-I activity and childhood-onset SLE subclinical atherosclerosis and endothelial function (150). We need a longitudinal assessment in the future to assess whether vascular damage in childhood-onset SLE is related to IFN-I. Inflammatory body activation is a key downstream pathway leading to vascular abnormalities. The interaction between IFN-I and inflammatory factors mediates reduced number and impaired function of SLE EPCs. IFN-α down-regulates IL-1β and VEGF (52) and up-regulates IL-18 and its activator caspase-1 (51)— IL-1β promotes the differentiation of EPCs (52); IL-18 inhibits the differentiation of EPCs (51). IL10 inhibits EC differentiation and enhances IFN-α-mediated EPC dysfunction (50). OPG plays a pathogenic role in atherosclerosis. OPG binds to syndecan 4, the receptor of OPG on EPC, then induces oxidative stress, causing apoptosis of EPC (151). Spinelli FR et al. has observed that BAFF receptors are expressed in both EPC and EC, and mediated the apoptosis of EPC (77). The addition of BAFF inhibitor—belimumab—restored the quantity and quality of EPCs *in vivo* and *in vitro*, which further proved this point (77).

Tang, a specific T cell group expressing CD3, CD31 and CXCR4, promotes early EPCs differentiation and activates locally resident ECs (161). And the percentage of circulating Tang increased in SLE patients (162–164). However, the chronic inflammatory environment of SLE accelerates autoimmune aging. Aging Tang (CD28null-Tang) is not protective but cytotoxic, secreting inflammatory mediators and releasing cytolytic molecules from intracellular particles to induce EC damage and accelerates atherosclerosis in most SLE patients (165). And the frequency of CD28null-Tang increased in SLE patients with traditional CVD risk factors and active diseases (165).

Therefore, we speculate that Tang activates the vascular endothelial protective mechanism in the early SLE. With the progress of the disease, the chronic inflammatory environment of SLE not only accelerates the aging of Tang but also enriches a variety of risk factors for EPCs, which leads to the dysfunction of EPC in SLE patients.

## The Role of IFN-I in the Injury of EPCs in SLE

### The Immune Mechanism of IFN-I Production in SLE

The IFN-I system in SLE is chronically active. pDCs (plasmacytoid pre-dendritic cells) are the primary source, which have high levels of interferon regulatory factor (IRF) 7, facilitating rapid and large-scale IFN-α generation (166). Up-regulated interferon-induced genes such as MX1, ISG54, and ISG56 and transcription factors of interferon pathway such as IRF5, IRF7, IRAK1, TREX1, STAT4, and PTPN22 mediate abnormal immune responses and the production of ICs, resulting in abnormal activation of pDCs (167). And other immune cells such as neutrophils, NK cells, T cells, B cells and platelets enhance IFN-I production by IC-stimulated pDCs; IFN-I, in turn, stimulates the activation of these immune cells, forming a self-magnifying pathogenic loop (65, 66, 168–173).

During exploring the signaling pathway, the increased exposure of nuclear contents to corresponding nucleic acid biosensors is the critical risk factors. Under normal physiological conditions, self DNA/RNA exists in different cell compartments and is isolated from the nucleic acid biosensor in the cytoplasm. Due to the insufficient clearance of apoptotic/necrotic cells, SLE patients are rich in endogenous free DNA/RNA, which form ICs with anti-DNA/RNA antibodies (174). Exogenous microbial DNA/RNA also induce autoimmune response (175–177). Exposed RNA and DNA stimulate the relevant nucleic acid biosensor in the form of ICs. DNA biosensors are divided into two types: endosomal membrane receptors and intracellular receptors (178). TLR9 is the only known DNA biosensor based on endosomes, which is mainly expressed in pDCs. The DNA ICs are absorbed and transported into the endosome through the Fcγ RIIa in pDCs, activating TLR9-MyD88-IRF7 pathway (166). Moreover, TLR9 can bind to the curli-DNA complex, composed of bacterial DNA and amyloid protein curli—a component of bacterial biofilms (175, 176). Compared with TLR9, cytoplasmic DNA biosensors are widely expressed in mammalian cells. Thirteen cytoplasmic DNA biosensors have been found so far and cGAS is the most important cytoplasmic DNA biosensor (178). cGAS binds to cytoplasmic DNA to produce cGAMP, which then activates ER-resident STING protein. The activated STING is transported from the endoplasmic reticulum to the ER-resident Golgi apparatus and recruits TBK1 to enter the endosome. TBK1 activates IRF3 and IRF7, leading to the expression of IFN-I (179). Major RNA biosensors include TLR7 and RIG-I/MDA5. TLR7 also belongs to the endosomal membrane receptor, activated by single-stranded RNA. The U1snRNA induces PDCs to produce IFN-α through Fcγ RIIa-TLR7-MyD88-IRF7 pathway in SLE patients (180, 181). RIG-I/MDA5 signal is mainly used to deal with viral infections. After recognizing viral double-stranded RNA, intracellular RNA helicases (RIG-I and MDA5) undergo conformational changes to induce MAVS, and activates IRF3/7 through TRAF6/3, resulting in the secretion of IFN-I (182). Recent studies have shown that RIG-I/MDA5 signal may reduce the degradation capacity of insoluble virus-like aggregates, inducing a continuous increase of IFN-I (177).

### The Pathways of IFN-I Damaging EPCs

**Figure 4:**
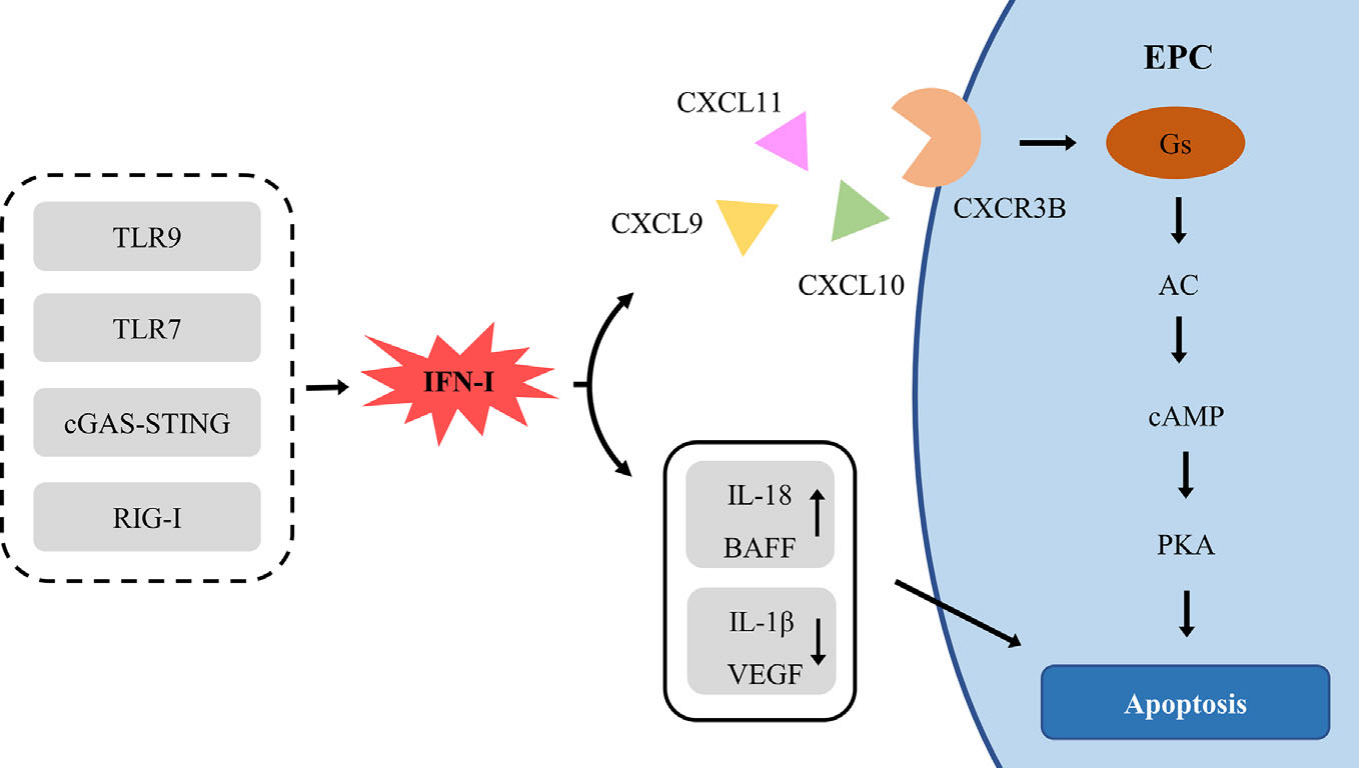
The signal pathway of IFN-Idamaging EPC. AC, adenylyl cyclase; BAFF, B cell-activating factor; cAMP, cyclic adenosine monophosphate; cGAS, cyclic guanosine monophosphate (GMP)-adenosine monophosphate (AMP) synthase; CXCL, chemokine (C-X-C Motif) ligand; CXCR, chemokine (C-X-C motif) receptor; EPC, endothelial progenitor cell; PKA, Protein kinase A; RIG-I, retinoic acid-inducible gene I; TLR, Toll-like receptor; VEGF, vascular endothelial growth factor.

IFN-I is one of the causes of impaired EPCs, but the specific mechanism remains to be elucidated. IFN-I damages EPCs in two ways: direct toxicity and indirect toxicity (**Figure 4**).

IFN-I actively induces the production of ELR-negative CXC chemokines CXCL9, CXCL10 and CXCL11, which mediate angiostasis through the receptor CXCR3 (183). CXCR3 exists in three different splice variants, CXCR3A, CXCR3B, and CXCR3-alt (184). CXCR3A recruits leukocytes, especially in Th1 lymphocytes (185). CXCR3-alt has a higher affinity for CXCL11, but its role in angiogenesis remains to be determined (186). Conversely, CXCR3B, expressed in ECs, is the main angiostatic variant of CXCR3 and is the primary angiostatic receptor for CXCL9, CXCL10, and CXCL11, inducing anti-proliferation and anti-migration (187–189). CXCR3A and CXCR3B differ for 52 amino acids at the NH2 end and couple different types of G proteins, triggering different signal transduction pathways, CXCR3A-Gi-PI3K-MAPK and CXCR3B-Gs-AC-cAMP-PKA (187, 190). The coupling of CXCR3B with Gs results in the selective activation of adenylyl cyclase (AC) and a consequent increase of intracellular cAMP levels (187). Up-regulation of cAMP in ECs directly activates PKA, inducing apoptosis (191).

Moreover, IFN-I enhances the toxicity of ILs and BAFF, which are EPC risk factor as well. IFN-I interacts with inflammatory factor ILs to damage EPC synergistically. IL-10 enhances the effect of IFN-α on SLE EPC (50). IFN-I down-regulates angiogenic molecules IL-1β and VEGF (52) and up-regulates IL-18 and its activator caspase-1 (51), enhancing the anti-angiogenic effect. There was a positive correlation between the levels of IFN-I and BAFF in SLE (192). IFN-I induces the expression and mobilization of BAFF in SLE monocytes and neutrophils (35, 36). The expression of BAFF is directly induced by IFN-I through IRF1 and IRF2 (36). IFN-α stimulates the secretion of IL-17, then IL-17 and BAFF promote the survival and differentiation of B cells and production of autoantibodies, which enhances IFN by pDCs, forming a closed vicious circle (192).

Therefore, IFN-I has direct and indirect toxic effects on EPC, resulting in endothelial dysfunction, which starts atherosclerosis in SLE. It is proved once again that IFN-I plays a central pathogenic role in SLE CVD.

## Conclusion

Long-term activation of IFN-I system in SLE induces the expression of CXCL9/10/11, activating CXCR3B-Gs-AC-cAMP-PKA signal pathway to promote the dysfunction of ECs and EPCs; and CXCR3A-Gi-PI3K-MAPK signaling pathway to recruit leukocytes into the inflammatory site. Besides, IFN-I enhances the toxicity of other EPCs dysfunction factors, indirectly accelerating arteriosclerosis. Overexpression of IFN-I through the activation of TLR7/9 signal decreases the number and function of EPCs and increases atherosclerotic lesions in SLE patients (46), suggesting that targeted therapy of cGAS and RIG-I signal pathway may have a potential therapeutic effect on SLE atherosclerosis. Targeted therapy of the IFN-I system has a potential therapeutic effect on early atherosclerosis in SLE patients.

## Author Contributions

XD did the literature search and drafted the article. WX and XH gave insight. XH revised the article. All authors contributed to the article and approved the submitted version.

## Funding

This work was supported by National Natural Science Foundation of China (No. 61562021 and No. 81560275, No. 81960885, No. 81260139, No. 81060073, No. 30560161), Hainan Major Science and Technology Projects (ZDKJ2039010), Hainan Association for academic excellence Youth Science and Technology Innovation Program (201515), Hainan special projects of Social Development (ZDYF2018103 and 2015SF39). 

## Conflict of Interest

The authors declare that the research was conducted in the absence of any commercial or financial relationships that could be construed as a potential conflict of interest.
