# Supplementary material for: IFN-I Mediates Dysfunction of Endothelial Progenitor Cells in Atherosclerosis of Systemic Lupus Erythematosus
Source: Front Immunol. 2020 Nov 11;11:581385. doi: 10.3389/fimmu.2020.581385 (PMC7686511; doi:10.3389/fimmu.2020.581385)
Supplement: Supplementary file 1 [file DataSheet_1.pdf]

AAVE, anti-vascular endothelial-cadherin antibody; AC, adenylyl cyclase; AECA, anti-endothelial cell antibody; aPL, antiphospholipid antibody; BAFF, B cell-activating factor; BAFF-R, B cell-activating factor receptor; BMSC, bone marrow stromal cell; cAMP, cyclic adenosine monophosphate; cGAMP, cyclic guanosine monophosphate-adenosine monophosphate; cGAS, cyclic guanosine monophosphate-adenosine monophosphate synthase; CXCL， chemokine (C-X-C Motif) ligand; CXCR, chemokine (C-X-C motif) receptor; CVD, cardiovascular disease; dsRNA, double-stranded RNA; EC, endothelial cell; ECFC, endothelial colony forming cell; EPC, endothelial progenitor cell; eNOS, endothelial nitric oxide synthase; FAK, focal adhesion kinase; GC, germinal centre; HDAC3, histone deacetylase 3; HDL, high-density lipoprotein; HIF-1, hypoxia-inducible factor 1; HoxA9, Homeobox A9; IC, immune complex; ICAM-1, intercellular adhesion molecule-1; IFN, interferon; iNKT, invariant natural killer T cell; IRF, interferon regulatory factor; LPL, lipoprotein lipase; MAC, myeloid angiogenic cell; MCP-1, monocyte chemotactic protein-1; MDA, malondialdehyde; MDA5, melanoma differentiation-associated gene 5; MIF, macrophage migration inhibitory factor; mKitL, membrane-bound form of Kit ligand; MMP, matrix metalloproteinase; MPO, myeloperoxidase; mTOR, mammalian target of rapamycin; NET, neutrophil extracellular trap; NK, natural killer T cell; OPG, Osteoprotegerin; OxLDL, oxidized low-density lipoprotein; PC, choline phosphate; PCSK, Proprotein convertase subtilisin/Kexin; pDC, plasmacytoid pre-dendritic cell; piHDL, proinflammatory high-density lipoprotein; PI3K, phosphatidylinositol-3-kinase; PKA, Protein kinase A; RIG-I，retinoic acid-inducible gene I; ROS, reactive

oxygen species; SDF-1, stromal cell-derived factor-1; sKitL, soluble Kit-ligand; SLE, systemic lupus erythematosus; ssRNA, single-stranded RNA; Tang, Angiogenic T cells; TFH, follicular helper T cell; TFPI, tissue factor pathway inhibitor; TLR, Toll-like receptor; TPF, peripheral helper T cell; TREX1, three-prime repair exonuclease; VCAM-1, vascular cell adhesion molecule-1; VEGF, vascular endothelial growth factor; VEGFR, vascular endothelial growth factor receptor; VSMC, vascular smooth muscle cell.
